# Home Blood Pressure Can Predict the Risk for Stroke/Bleeding Events in Elderly Patients With Nonvalvular Atrial Fibrillation From the ANAFIE Registry

**DOI:** 10.1161/HYPERTENSIONAHA.122.19810

**Published:** 2022-10-19

**Authors:** Kazuomi Kario, Naoyuki Hasebe, Ken Okumura, Takeshi Yamashita, Masaharu Akao, Hirotsugu Atarashi, Takanori Ikeda, Yukihiro Koretsune, Wataru Shimizu, Shinya Suzuki, Hiroyuki Tsutsui, Kazunori Toyoda, Atsushi Hirayama, Masahiro Yasaka, Takenori Yamaguchi, Satoshi Teramukai, Tetsuya Kimura, Yoshiyuki Morishima, Atsushi Takita, Hiroshi Inoue

**Affiliations:** Jichi Medical University, Tochigi, Japan (K.K.).; Asahikawa Medical University, Hokkaido, Japan (N.H.).; Division of Cardiology, Saiseikai Kumamoto Hospital Cardiovascular Center, Kumamoto, Japan (K.O.).; The Cardiovascular Institute, Tokyo, Japan (T.Yamashita, S.S.).; Department of Cardiology, National Hospital Organization Kyoto Medical Center, Kyoto, Japan (M.A.).; AOI Hachioji Hospital, Tokyo, Japan (H.A.).; Department of Cardiovascular Medicine, Toho University Faculty of Medicine, Tokyo, Japan (T.I.).; National Hospital Organization Osaka National Hospital, Osaka, Japan (Y.K.).; Department of Cardiovascular Medicine, Graduate School of Medicine, Nippon Medical School, Tokyo, Japan (W.S.).; Department of Cardiovascular Medicine, Faculty of Medical Sciences, Kyushu University, Fukuoka, Japan (H.T.).; Department of Cerebrovascular Medicine, National Cerebral and Cardiovascular Center, Osaka, Japan (K.T., T.Y amaguchi).; Osaka Police Hospital, Osaka, Japan (A.H.).; Department of Cerebrovascular Medicine and Neurology, National Hospital Organization Kyushu Medical Center, Fukuoka, Japan (M.Y.).; Department of Biostatistics, Graduate School of Medical Science, Kyoto Prefectural University of Medicine, Kyoto, Japan (S.T.).; Daiichi Sankyo, Tokyo, Japan (T.K., Y.M., A.T.).; Saiseikai Toyama Hospital, Toyama, Japan (H.I.).

**Keywords:** atrial fibrillation, blood pressure, elderly, embolism, hemorrhage

## Abstract

Blood pressure (BP) fluctuates significantly in patients with atrial fibrillation (AF); office BP measurements seem insufficient to assess AF patient risk accurately. We hypothesized that home BP could better predict the risk of stroke/systemic embolic events (SEE) and major bleeding in patients with AF than office BP.

**Methods:**

In this prespecified subcohort study of the ANAFIE (All Nippon AF in the Elderly) Registry, we evaluated the impact of home BP on the risk of stroke/SEE, major bleeding, intracranial hemorrhage, all-cause death, and net cardiovascular outcome (a composite of stroke/SEE and major bleeding). At enrollment, home BP was measured twice in the morning and evening for 7 days.

**Results:**

In total, 4933 elderly patients (aged ≥75 years) with nonvalvular AF participated. Incidences of net cardiovascular outcome, stroke/SEE, major bleeding, and intracranial hemorrhage increased significantly with increasing home systolic BP (H-SBP). Compared with H-SBP <125 mm Hg, ≥145 mm Hg was associated with increased risk of these events. The association between H-SBP and the events was observed only in patients with ≥20 H-SBP measurements.

**Conclusions:**

In elderly patients with nonvalvular AF, high H-SBP (≥145 mm Hg) was a significant predictor of stroke/SEE, major bleeding, and intracranial hemorrhage risk. Strict BP control guided by the increasing number of home BP measurements may provide an accurate clinical outcome risk assessment.

**Registration:**

URL: https://www.umin.ac.jp/ctr; Unique identifier: UMIN000024006

**Novelty and Relevance**

**What Is New?**

Compared with home systolic blood pressure (BP) <125 mm Hg, home systolic BP ≥145 mm Hg was associated with increased risk of stroke/systemic embolic events, major bleeding, and intracranial hemorrhage in elderly patients with nonvalvular atrial fibrillation (NVAF). Increases in these outcomes were not identified by office systolic BP.

**What Is Relevant?**

Office BP measurements may be insufficient for assessing risk in patients with nonvalvular atrial fibrillation, who experience considerable fluctuations in BP given the limited number of measurements taken in clinical practice and possible variability stemming from the white coat effect.
At a high number of home BP measurements (≥20) the predictive ability increases, indicating that increasing the number of BP readings is beneficial for determining risk.
Among elderly patients with nonvalvular atrial fibrillation, home systolic BP ≥145 mm Hg was a significant predictor of the risk of stroke/systemic embolic events, major bleeding, and intracranial hemorrhage.

**Clinical/Pathophysiological Implications?**

Compared with office BP, home BP is more useful in identifying at-risk patients. Therefore, strict blood pressure control guided by consecutive home BP measurements may provide a more accurate risk assessment.

Atrial fibrillation (AF) is a major risk factor of ischemic stroke, affecting the life expectancy of elderly patients.^1^ The incidence and prevalence of AF increase with age.^2,3^ The ANAFIE (All Nippon AF in the Elderly) Registry, a prospective, observational study, was conducted to clarify the real-world clinical status and prognosis of >30 000 elderly patients aged ≥75 years with nonvalvular AF (NVAF).^4^

**See editorial, pp 2706–2707**

Hypertension is an independent risk factor for stroke/systemic embolic events (SEE) and bleeding complications in patients with AF.^5^ Hypertension and AF can afflict the same patient.^6^ Hypertension is thought to amplify the risk of AF occurrence and thromboembolic disease via hemodynamic and nonhemodynamic mechanisms.^7^ Hypertension is a modifiable component of the CHADS_2_, CHA_2_DS_2_-VASc, and HAS-BLED (hypertension, abnormal liver/renal function, stroke history, bleeding history or predisposition, labile prothrombin time international normalized ratio, elderly, drug/alcohol usage) scores.^8^ The BAT (Bleeding With Antithrombotic Therapy) Study showed that an increase in blood pressure (BP) is associated with a higher risk of intracranial hemorrhage (ICH),^9^ and guidelines recommend controlling office BP (O-BP) below 130 mm Hg.

Several factors should be considered when evaluating the effect of BP on the risk of cardiovascular events and prognosis in very elderly patients with AF. Systolic BP (SBP) and diastolic BP (DBP) fluctuate significantly in patients with AF compared with patients with sinus rhythm.^10^ The limited number of O-BP measurements performed in current clinical practice is insufficient for accurately assessing BP risk in patients with AF. The strength of the association between BP and cardiovascular disease risk is significantly different among age groups. In very elderly patients (75–89 years), the impact of elevated BP on cardiovascular disease risk becomes less obvious.^11^

We hypothesized that home BP (H-BP) would be better at predicting the risk of stroke/SEE and major bleeding in patients with AF than O-BP, as this method allows for a greater number of measurements, which can overcome variability in patients with AF and avoids variability from the white coat effect.^12,13^ In this prespecified H-BP subcohort study of the ANAFIE Registry,^4,14^ we evaluated the impact of H-BP on the risk of stroke/SEE, major bleeding, ICH, all-cause death, and net cardiovascular outcome (a composite of stroke/SEE and major bleeding).

## Methods

The authors declare that all supporting data are available within the article and its Supplementary Materials.

### Study Design

The rationale and methodology of the ANAFIE Registry have been previously published.^14^ Ethical approvals were obtained from all relevant institutional review boards, the principal being the Ethics Committee of The Cardiovascular Institute (Tokyo, Japan). The trial was registered at University Hospital Medical Information Network (UMIN) Clinical Trials Registry. The study was conducted per the Declaration of Helsinki, local registry requirements, and ethical guidelines for clinical studies in Japan.^14^

### Patients

The main inclusion criteria were age ≥75 years at the time of informed consent, a definitive diagnosis of NVAF, and the ability to attend hospital visits. The only specific enrollment condition for the H-BP subcohort study was that patients provided written consent to be enrolled and agreed to measure their H-BP using an oscillometric device with an arm cuff.^15,16^ The main exclusion criteria are listed in the Supplementary Methods. All participants gave informed consent for the main ANAFIE Registry and this subcohort study and were free to withdraw at any time.^16^

### Study End Points

The study endpoints were net cardiovascular outcome (ie, composite of stroke/SEE and major bleeding), stroke/SEE, major bleeding, ICH, and all-cause death. These events that occurred during the follow-up period were recorded in duplicate. All endpoint events were adjudicated by cerebrovascular, cardiac, and bleeding event evaluation committees consisting of neurologists, cardiologists, and hematologists. Major bleeding was classified per the International Society on Thrombosis and Haemostasis definition (Supplementary Methods).^17^

### Data Collection

The H-BP and O-BP measurement procedures were based on those described by Kario et al.^16^ After receiving guidance on correctly and accurately conducting H-BP readings, patients measured their H-BP four times/day, twice in the morning and twice in the evening, for 1 week within 60 days of obtaining informed consent, per the Japanese Society of Hypertension guidelines for the management of hypertension (JSH 2019).^18^ Any device based on the brachial cuff oscillometric method was allowed. Values were registered in an H-BP recording sheet. H-BP measurements over 1 week were averaged and analyzed. O-BP measurements were the average of 2 measurements taken on the closest visit date following the consent acquisition.

### Statistical Analysis

Detailed statistical analysis methods applied to the ANAFIE Registry data have been previously described.^4,14^

To clarify the impact of BP on stroke/SEE, major bleeding, ICH, all-cause death, and net cardiovascular outcome risk in this cohort, the Kaplan-Meier method was used to estimate event incidence and to illustrate event occurrence. BP categories were chosen per the JSH 2019 guidelines.^18^ A Cox proportional hazards model was used to calculate hazard ratios (HRs) and corresponding 95% CIs for event risk to evaluate the effect of BP. To evaluate the accuracy of home SBP (H-SBP) versus office SBP (O-SBP) in predicting event risk in patients with NVAF, given the tendency for patients with AF to experience fluctuations in BP, a likelihood ratio test was used to assess improvements in the goodness-of-fit model for each event of interest.^19^ The Akaike Information Criterion and Schwarz Bayesian Information Criterion were calculated. An additional analysis was performed using the SBP categories outlined in the American Heart Association 2017 guideline.^20^ *P* values for the incidence rate ratio (relative risk) were calculated using the Poisson regression model. A spline regression analysis was performed as described in Supplementary Methods. Statistical tests were 2-sided, and the significance level was defined as *P*<0.05. All statistical analyses were performed using SAS version 9.4 (SAS Institute, Tokyo, Japan).

## Results

### Patient Characteristics

From the total ANAFIE Registry population, 4933 patients participated in this subcohort study; 56.2% were men, and the average age was 81.4 years. The mean CHA_2_DS_2_-VASc and HAS-BLED scores were 4.4 and 1.8, respectively; 77.6% of patients had hypertension, and 93.0% used anticoagulants. The mean BP values were 127.8±13.1 mm Hg for H-SBP, 72.6±9.1 mm Hg for home DBP (H-DBP), 128.4±17.2 mm Hg for O-SBP, and 71.3±11.5 mm Hg for office DBP. The mean number of measurements was 24.7±6.2 for H-BP and 2 for O-BP. During the 2-year follow-up period, 115 stroke/SEE and 76 major bleeding events were reported (Table 1). Table S1 shows the main characteristics of patients enrolled in the overall ANAFIE Registry and this H-BP subcohort.

**Table 1.**
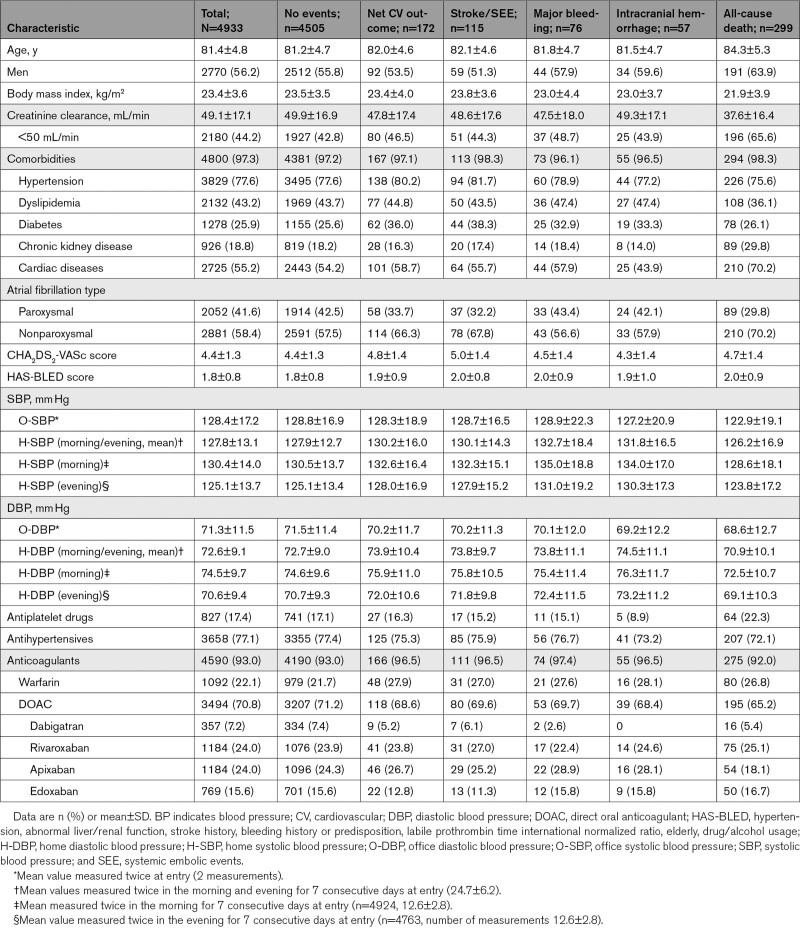
Background Characteristics of Patients With No Events, Stroke/SEE, or Major Bleeding Events

### Systolic BP Analysis

H-SBP was categorized according to the JSH 2019 guidelines.^18^The H-SBP category with the most patients was <125 mm Hg (n=2030 [41%]) followed by 125 to <135 mm Hg (n=1585 [32%]), 135 to <145 mm Hg (n=878 [18%]), and ≥145 mm Hg (n=440 [9%]).

**Figure 1.**
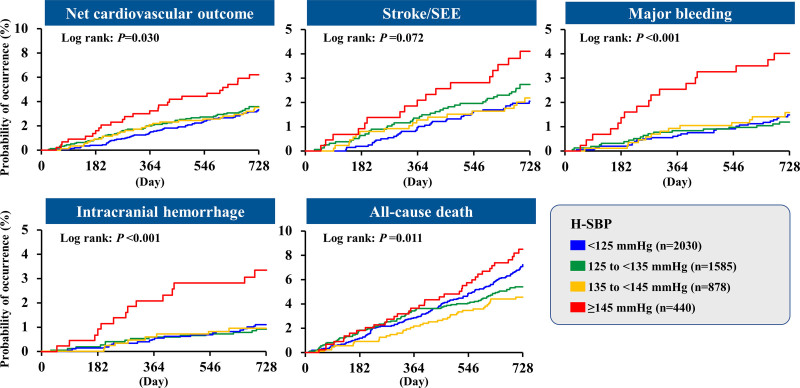
Kaplan–Meier curves of net cardiovascular outcome, stroke/systemic embolic events (SEE), major bleeding, intracranial hemorrhage, and all-cause death according to home systolic blood pressure (H-SBP).

In general, increases in these events were observed with increasing H-SBP and were significant for all outcomes except for stroke/SEE. All-cause death was lowest among patients in the 135 to <145 mm Hg group; the highest probability of occurrence was in the ≥145 mm Hg group (Figure 1).

**Figure 2.**
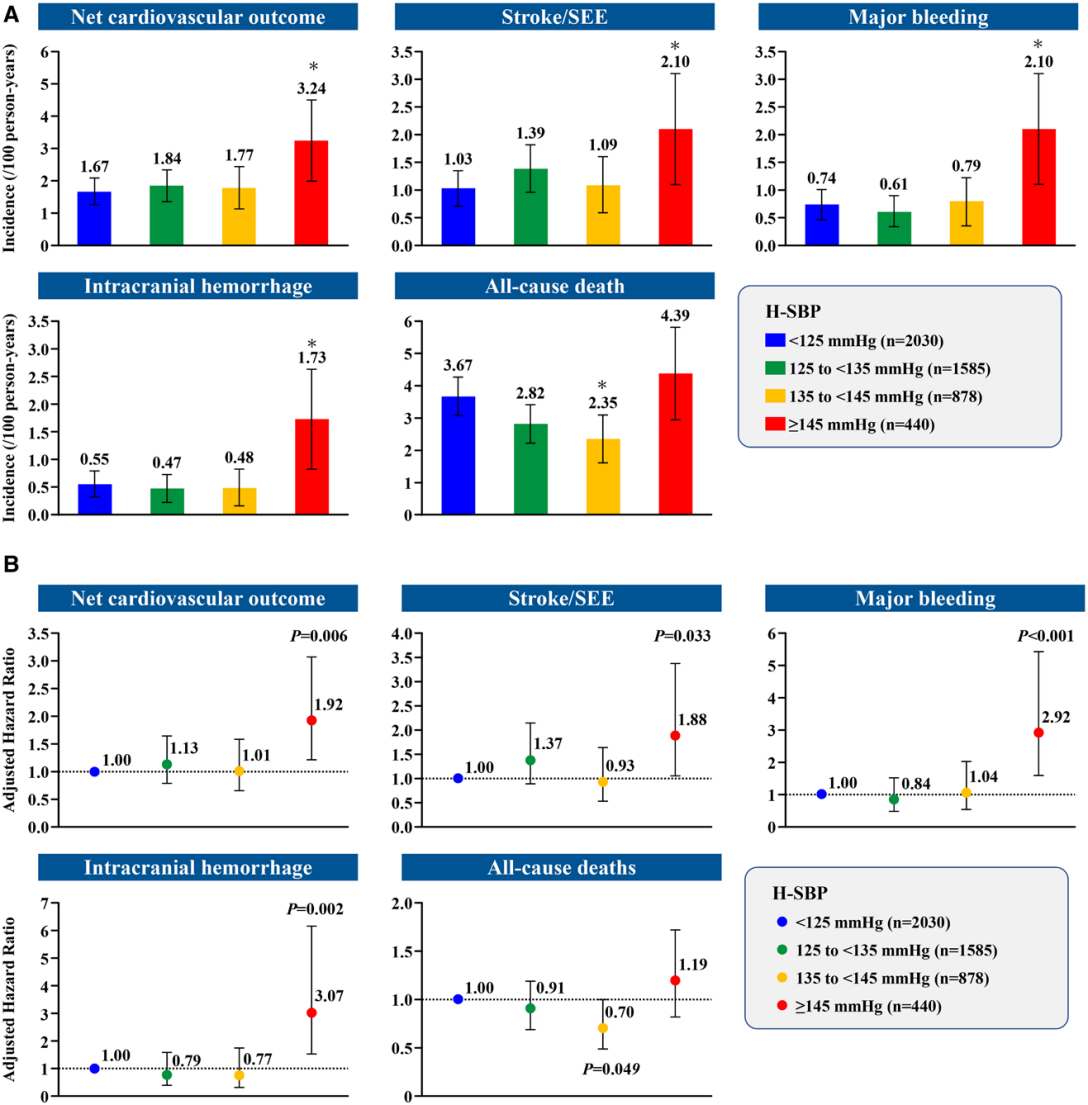
**Incidence rates of clinical outcomes in patients with atrial fibrillation according to home systolic blood pressure (H-SBP). A**, Incidence rates of net cardiovascular outcome, stroke/systemic embolic events (SEE), major bleeding, intracranial hemorrhage, and all-cause death according to H-SBP. Bars represent 95% CIs. *P<0.05 versus H-SBP <125 mm Hg. **B**, Influence of H-SBP on net cardiovascular outcome, stroke/SEE, major bleeding, intracranial hemorrhage, and all-cause death. The Cox proportional hazards model was used, adjusted by sex, age, body mass index, history of major bleeding, atrial fibrillation type, severe hepatic dysfunction, diabetes, hyperuricemia, heart disease (heart failure, left ventricular systolic dysfunction), myocardial infarction, cerebrovascular disease, thromboembolic-related disease, active cancer, dementia, falls within 1 year, anticoagulants, nonpharmacologic therapy (catheter ablation), antiarrhythmics, antiplatelet agents, proton pump inhibitors, P-glycoprotein inhibitors, dyslipidemia, creatinine clearance, gastrointestinal disease, and polypharmacy. Bars represent 95% CIs.

Among patients in the <125 mm Hg group and the ≥145 mm Hg group, the respective incidence rates per 100 person-years were 1.67 and 3.24 (*P*<0.05) for net cardiovascular outcome, 1.03 and 2.10 for stroke/SEE (*P*<0.05), 0.74 and 2.10 for major bleeding (*P*<0.05), 0.55 and 1.73 for ICH (*P*<0.05), and 3.67 and 4.39 for all-cause death (*P*=0.337; Figure 2A).

Compared with H-SBP <125 mm Hg, H-SBP ≥145 mm Hg was significantly associated with higher incidences of net cardiovascular outcome (HR, 1.92 [95% CI, 1.21–3.06]; *P*=0.006), stroke/SEE (HR, 1.88 [95% CI, 1.05–3.37]; *P*=0.033), major bleeding (HR, 2.92 [95% CI, 1.58–5.42]; *P*<0.001), and ICH (HR, 3.07 [95% CI, 1.54–6.15]; *P*=0.002). There were no significant associations in other H-SBP categories (≥125 mm Hg to <135 mm Hg, ≥135 mm Hg to <145 mm Hg) for these outcomes. For all-cause death, H-SBP 135 to <145 mm Hg was significantly associated with a lower incidence compared with H-SBP <125 mm Hg (HR, 0.70 [95% CI, 0.49–1.00]; *P*=0.049; Figure 2B).

**Figure 3.**
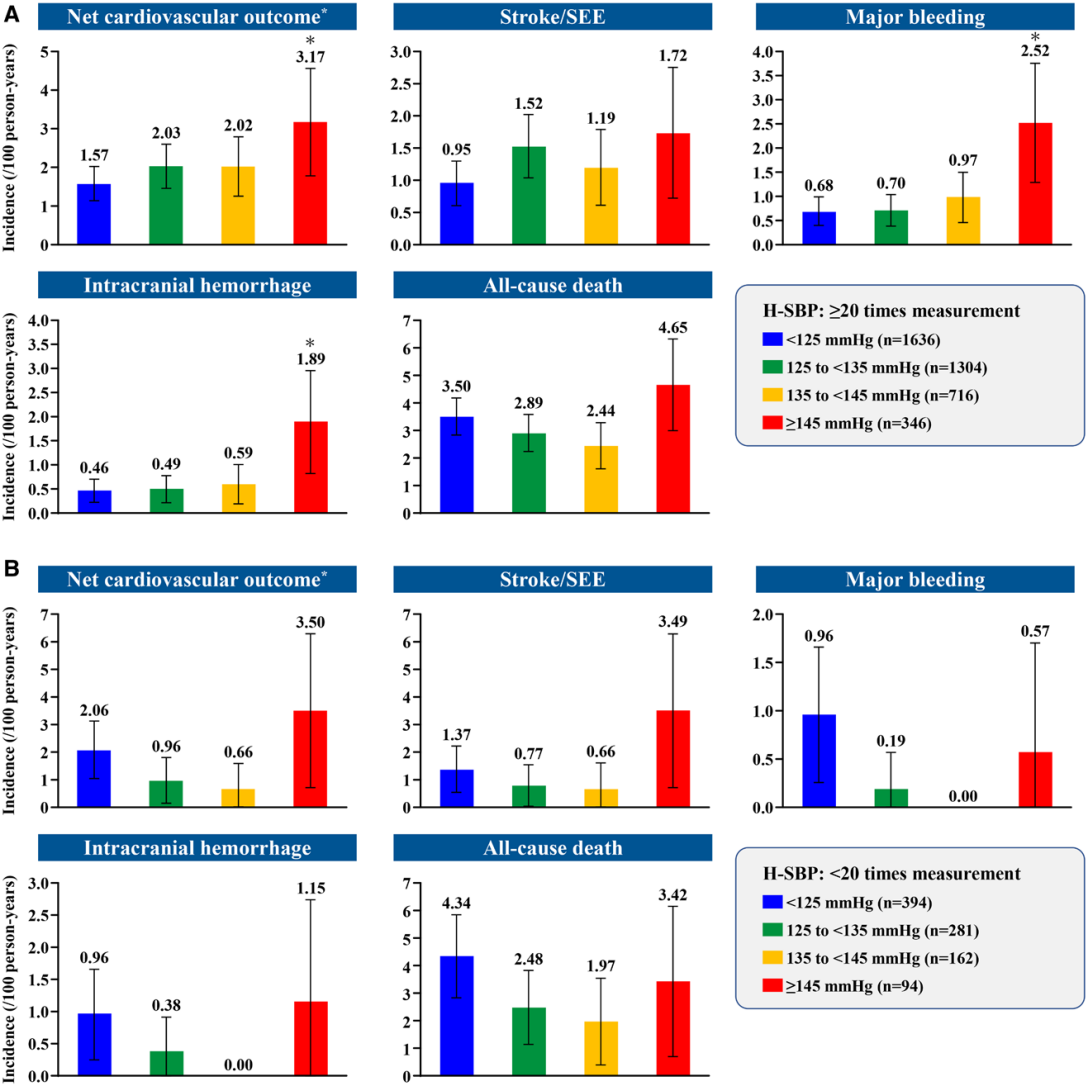
**Home systolic blood pressure (H-SBP) and incidence rates of clinical outcomes in patients with atrial fibrillation according to number of H-SBP measurements. (A**) ≥20 and (**B**) <20 H-SBP. *P<0.05 versus home systolic blood pressure<125 mm Hg.

In patients with ≥20 H-SBP measurements, the incidence rates of net cardiovascular outcome, stroke/SEE, major bleeding, and ICH significantly increased in the H-SBP ≥145 mm Hg group compared with the <125 mm Hg group, but not all-cause death. Even in patients with <20 H-SBP measurements, the incidence rate of stroke/SEE was numerically higher at H-SBP ≥145 mm Hg. There were no significant differences in clinical outcomes, including stroke/SEE, between the H-SBP ≥145 mm Hg and <125 mm Hg groups (Figure 3).

Regarding O-SBP, none of the BP categories were significantly associated with increased risk of any events assessed (Figure S1). The goodness-of-fit of the models for predicting risks of the net cardiovascular outcome, major bleeding, and ICH were significantly improved by adding H-SBP, but not O-SBP, to the model, including only confounders (Table 2). Similar results were shown by Akaike Information Criterion and Schwarz Bayesian Information Criterion (Table S2).

**Table 2.**
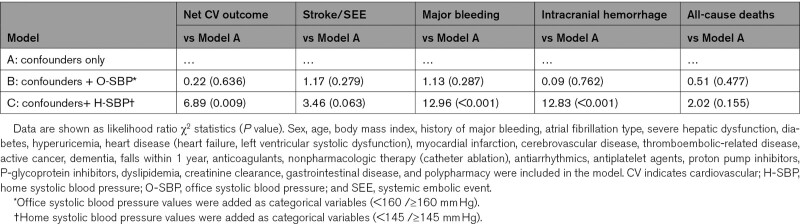
Improvement in Goodness-of-fit Model for Net CV Outcome, Stroke/SEE, Major Bleeding, Intracranial Hemorrhage, and All-cause Death by Addition of Categorical Variables of H-SBP (<145 mm Hg/≥145 mm Hg) or O-SBP (<160 mm Hg/≥160 mm Hg)

### Home Diastolic BP Analysis

H-DBP was also categorized according to the JSH 2019 guidelines.^18^ The incidence rates of net cardiovascular outcome, stroke/SEE, major bleeding, ICH, and all-cause death tended to increase with increasing H-DBP. Significant differences were observed for all outcomes among patients with H-DBP ≥90 mm Hg compared with <75 mm Hg, except for all-cause death (Figure S2).

Cox multivariate regression analysis also showed that compared with H-DBP <75 mm Hg, H-DBP ≥90 mm Hg was significantly associated with higher incidences of net cardiovascular outcome (HR, 3.17 [95% CI, 1.82–5.52]; *P*<0.001), stroke/SEE (HR, 2.46 [95% CI, 1.16–5.20]; *P*=0.019), major bleeding (HR, 4.03 [95% CI, 1.85–8.76]; *P*<0.001), and ICH (HR, 4.79 [95% CI, 2.03–11.28]; *P*<0.001; Figure S3). H-DBP 75 to <85 mm Hg was significantly associated with a lower incidence for all-cause death (HR, 0.72 [95% CI, 0.54–0.95]; *P*=0.020).

### Additional Analysis

Per the American Heart Association 2017 guideline,^20^ the incidence rates of net cardiovascular outcome, stroke/SEE, major bleeding, and ICH were significantly increased in the H-SBP ≥145 mm Hg group compared with the <130 mm Hg group (Figure S4).

In the spline regression analysis, the H-SBP value for minimum risks of net cardiovascular outcome and major bleeding was 118 mm Hg. The H-SBP values at which the lower limit of the 95% CI exceeded a relative risk of 1 were 143 and 142 mm Hg, respectively (Figure S5).

Table S3 shows the incidence rates by type of hypertension. Masked hypertension (defined as H-SBP ≥145 mm Hg/O-SBP <160 mm Hg) was associated with significantly higher incidence rates of net cardiovascular outcome, stroke/SEE, major bleeding, and ICH. Sustained hypertension (H-SBP ≥145 mm Hg/O-SBP ≥160 mm Hg) was associated with an increased risk of major bleeding. However, in the white coat hypertension (H-SBP <145 mm Hg/O-SBP ≥160 mm Hg) group, there was no increase in the incidence rates of clinical outcomes. As a result of setting the type of hypertension with different criteria of SBP, sustained hypertension (defined as H-SBP ≥135 mm Hg/O-SBP ≥140 mm Hg) was associated with significantly higher incidence rates of net cardiovascular outcome, major bleeding, and ICH (Table S4). Table S5 shows the causes of death reported in the subcohort study by H-SBP category.

## Discussion

This is the first large-scale real-world study of H-BP to show the importance of H-BP measurements in patients with NVAF. The incidence rates of net cardiovascular outcome, stroke/SEE, major bleeding, ICH, and all-cause death increased with increasing H-SBP, particularly H-SBP ≥145 mm Hg in elderly patients with NVAF. A significant association between high H-SBP and the increased risk of clinical outcomes was observed only in patients with ≥20 H-SBP measurements. However, there was no correlation between the risk of any outcomes assessed in this study with O-SBP. Additionally, the goodness-of-fit analysis showed that adding H-SBP to the model significantly improved the risk prediction of net cardiovascular outcome, major bleeding, and ICH, but not O-SBP. Furthermore, this study showed that H-DBP ≥90 mm Hg was a significant risk factor for net cardiovascular outcome, stroke/SEE, major bleeding, and ICH.

In hypertensive patients, H-BP is a known risk factor for cardiovascular remodeling and cardiovascular events.^12,13^ Japanese and other guidelines^18,20^ recommend hypertension treatment using H-BP as a guide. In HONEST (Home blood pressure measurement with Olmesartan Naive patients to Establish Standard Target blood pressure) and J-HOP (Japan Morning Surge-Home Blood Pressure),^19,21^ the total cardiovascular risk of hypertensive patients was predicted more strongly by H-BP than O-BP. Of note, the definitions of net outcome vary across studies. This study focused on major clinical events (stroke/SEE and bleeding) to determine the benefit of anticoagulant therapy and did not include all-cause death as a net cardiovascular outcome.

In AF, O-BP might not be an accurate BP assessment, given the high beat-to-beat BP variability.^10^ H-BP measurements may have the advantage of better reflecting actual BP, unaffected by phenomena such as the white coat effect.^22^ Furthermore, multiple measurements provide an average value that can overcome the BP variability in patients with AF and enable reliable risk assessment. Indeed, this analysis showed that ≥20 H-SBP measurements significantly detected the risk of events such as major bleeding, ICH, and net cardiovascular outcomes in patients with H-SBP ≥145 mm Hg. When the number of H-BP measurements was low (<20 measurements), the predictive ability decreased (ie, no significant association was observed between the incidence of events and BP categories), indicating that increasing the number of BP readings is beneficial for determining risk. Where patients with AF cannot provide reliable H-BP readings, other approaches, such as a higher number of O-BP readings or ambulatory BP monitoring, could be considered.

Cox multivariate regression and spline regression analyses showed a similar threshold value of about 145 mm Hg for the risks of net cardiovascular outcome and major bleeding, and the spline analysis indicated that the risks were minimum at 118 mm Hg. H-DBP ≥90 mm Hg was also a significant risk factor for net cardiovascular outcome, stroke/SEE, major bleeding, and ICH. The number of patients with H-DBP ≥90 mm Hg was small (n=164, 3.3%), and 48% of them were included in the H-SBP ≥145 mm Hg group. Similar results were obtained using H-SBP categories based on JSH 2019 and American Heart Association 2017 guidelines.^18,20^ This H-SBP of 145 mm Hg corresponds with the O-SBP of 160 mm Hg in the JSH 2019, NICE guidelines, and other guidelines.^18,20,23^ The ANAFIE population is a high-risk group of elderly patients with AF; their cardiovascular risk was higher than that of patients in the HONEST and J-HOP studies,^19,21^ which included almost no patients with AF. However, high H-BP was also a clear risk, with an abnormal threshold of H-SBP 145 mm Hg for stroke and bleeding. Therefore, it is meaningful to promptly control H-SBP to <145 mm Hg even for elderly (≥75 years) patients with AF.

In ANAFIE, O-SBP was not associated with the risk of net cardiovascular outcome, stroke/SEE, major bleeding, ICH, or all-cause death. Conversely, the Fushimi AF Registry (mean age 72 years) indicated that hypertension at baseline (O-SBP ≥150 mm Hg) is significantly associated with higher incidences of both stroke/SEE and major bleeding versus no hypertension.^24^ The J-RHYTHM (Japanese Rhythm Management Trial for Atrial Fibrillation) Registry (mean age 69.8 years) reported that O-SBP at the time closest to the event or at the end of follow-up (≥136 mm Hg) appears to be more important than a history of hypertension and baseline O-SBP values at preventing thromboembolism and major bleeding in patients with NVAF.^25^

In our study, O-BP was not associated with stroke/SEE risk or other endpoints, possibly because of the masking effects of aging among the very elderly. The main analysis of ANAFIE Registry (N=32 275), reported an increased risk of stroke/SEE, but not major bleeding or all-cause death among patients with O-SBP ≥140 mm Hg compared with <130 mm Hg (HR, 1.31; *P*=0.001).^4^ The difference in the effects of O-SBP on the risk of stroke/SEE between the main analysis of ANAFIE Registry and this subcohort study is unclear because there were no obvious differences in the baseline patient characteristics (Table S1). There might have been a selection bias in this subcohort study as 4933 out of 32 275 patients gave informed consent after it was explained to them that they were required to take an H-BP measurement each day for 1 week, suggesting that patients with a high level of health literacy might have chosen to participate in this subcohort study.

The BAT study, including patients with cardiovascular and cerebrovascular diseases (with or without AF) found that increased SBP and DBP during the study increased the risk of developing ICH; the estimated BP cutoff to predict ICH was ≥130/81 mm Hg.^9^ The strength of the association between BP and cardiovascular disease risk is significantly different among age groups. In very elderly patients (75–89 years), the impact of elevated BP on cardiovascular disease risk becomes less obvious^11^ because the risks associated with aging itself mask the risks associated with increased BP. Furthermore, because white coat hypertension increases in elderly patients,^16^ it becomes difficult to find a relationship between O-BP and cardiovascular risk. However, it should be noted that our study clarified the independent association between H-SBP at ≥145 mm Hg and the increased risks of net cardiovascular outcome, stroke/SEE, major bleeding, and ICH, even in the elderly patients with NVAF aged ≥75 years, by analyzing a large number of H-BP measurements.

All-cause death is an important endpoint for elderly patients. The incidence rate of all-cause death among patients in the <125 mm Hg group and the ≥145 mm Hg group (3.67 and 4.39/100 person-years, respectively) was high compared with other endpoints. A significant difference was observed between patients with H-SBP of <125 mm Hg versus 135 mm Hg to <145 mm Hg (incidence rate, 3.67 versus 2.35; HR, 0.70 [95% CI, 0.49–1.00]; *P*=0.049). The spline analysis of this event showed a J-curve pattern, which differed from other events. Regarding the cause of death, there was no remarkable difference in the incidence rate of cardiovascular death between H-SBP of <125 mm Hg versus 135 mm Hg to <145 mm Hg (incidence rate, 0.97 versus 0.78). Therefore, noncardiovascular death may contribute to the low incidence of all-cause death in the H-SBP 135 mm Hg to <145 mm Hg group. The findings from the current study differ from those of the SPRINT (Systolic Blood Pressure Intervention Trial) study.^26^ The SPRINT study reported that in patients with hypertension aged ≥75 years, treating SBP to a target of ≤120 mm Hg compared with a target of ≤140 mm Hg significantly reduced the incidence of all-cause death.^26^ Main reasons for this difference were that all patients in the current ANAFIE Registry had AF, whereas the SPRINT study enrolled patients with hypertension; the ANAFIE Registry is an observational study without BP targets, whereas the SPRINT study is a clinical trial in which patients were randomized by BP targets; and in the ANAFIE Registry, many patients associated with a high risk of death, such as frailty (36.2%) with low BP (125.5/68.9 mm Hg), were enrolled.^27^

The main study limitations have been reported^4^; here, we list those specific to this subcohort study. Changes in H-BP during the follow-up period were not examined, and the follow-up period was relatively short; however, future studies using H-BP measurements within longer follow-up may be helpful for accurate risk assessment. O-BP was the average of two measurements obtained at one visit; a future study analyzing more O-BP readings may predict clinical outcomes risk in patients with NVAF. Patients did not use the same BP device for H-BP measurements; using a standard device may lead to less measurement bias. Selection bias was possible, but there was no significant difference in patient background in the present subcohort study versus the overall ANAFIE Registry population. The relative impact of a single risk factor decreases with advancing age; thus, these findings should be confirmed in a relatively younger patient population with NVAF. Only Japanese patients were included, but future studies should evaluate the relationship between H-BP and clinical outcomes in non-Japanese patients with NVAF aged ≥75 years.

## Perspectives

For elderly patients with NVAF aged ≥75 years, more than 93% were on anticoagulant therapy in the ANAFIE Registry, H-SBP ≥145 mm Hg was significantly associated with an increase in the risk of net cardiovascular outcome, stroke/SEE, major bleeding, and ICH. O-SBP did not identify these increases in event risk. A significant association between H-SBP and clinical outcomes was observed only in patients with ≥20 H-SBP measurements. H-BP measurement was useful in identifying at-risk patients; therefore, strict BP control guided by an increased number of H-BP measurements should be recommended for elderly patients with NVAF.

## Article Information

### Acknowledgments

The authors wish to thank all individuals (physicians, nurses, institutional staff, and patients) involved in the ANAFIE (All Nippon AF in the Elderly) Registry. They also thank IQVIA Services Japan K.K. and EP-CRSU for their partial support in the conduct of this Registry, and Keyra Martinez Dunn, MD, of Edanz (www.edanz.com) for providing medical writing support, which was funded by Daiichi Sankyo Co, Ltd, in accordance with Good Publication Practice (GPP3) guidelines (http://www.ismpp.org/gpp3). In addition, the authors thank Daisuke Chiba of Daiichi Sankyo Co, Ltd, for support in the preparation of the article.

### Sources of Funding

This research was supported by Daiichi Sankyo Co., Ltd.

### Disclosures

K. Kario received research grants from Bristol Myers Squibb, Bayer, Daiichi Sankyo, Omron Healthcare, Inc, and A&D, Inc, and remuneration from Daiichi Sankyo, Bayer, and Omron Healthcare, Inc. N. Hasebe received research funding from Bristol Myers Squibb and Daiichi Sankyo, and remuneration from Daiichi Sankyo and Bayer. K. Okumura received remuneration from Nippon Boehringer Ingelheim, Daiichi Sankyo, Johnson & Johnson, and Medtronic. T. Yamashita received research funding from Bristol Myers Squibb, Bayer, and Daiichi Sankyo, manuscript fees from Daiichi Sankyo and Bristol Myers Squibb, and remuneration from Daiichi Sankyo, Bayer, Pfizer Japan, and Bristol Myers Squibb. M. Akao received research funding from Bayer and Daiichi Sankyo, and remuneration from Bristol Myers Squibb, Nippon Boehringer Ingelheim, Bayer, and Daiichi Sankyo. H. Atarashi received remuneration from Daiichi Sankyo. T. Ikeda received research funding from Daiichi Sankyo and Bayer, and remuneration from Daiichi Sankyo, Bayer, Nippon Boehringer Ingelheim, and Bristol Myers Squibb. Y. Koretsune received remuneration from Daiichi Sankyo, Bayer, and Nippon Boehringer Ingelheim. W. Shimizu received research funding from Bristol Myers Squibb, Daiichi Sankyo, and Nippon Boehringer Ingelheim, and patent royalties/licensing fees from Daiichi Sankyo, Pfizer Japan, Bristol Myers Squibb, Bayer, and Nippon Boehringer Ingelheim. S. Suzuki received research funding from Mitsubishi-Tanabe and Daiichi Sankyo, and remuneration from Bristol Myers Squibb and Daiichi Sankyo. H. Tsutsui received research funding from Daiichi Sankyo and Nippon Boehringer Ingelheim, remuneration from Daiichi Sankyo, Bayer, Nippon Boehringer Ingelheim, and Pfizer Japan, scholarship funding from Daiichi Sankyo, and consultancy fees from Pfizer Japan, Bayer, and Nippon Boehringer Ingelheim. K. Toyoda received lecture honoraria from Daiichi Sankyo, Otsuka, Novartis, Abbott, Bayer Yakuhin, and Bristol Myers Squibb outside the submitted work. A. Hirayama participated in a course endowed by Boston Scientific Japan, has received research funding from Daiichi Sankyo and Bayer, and remuneration from Bayer, Daiichi Sankyo, Bristol Myers Squibb, and Nippon Boehringer Ingelheim. M. Yasaka received research funding from Nippon Boehringer Ingelheim, and remuneration from Nippon Boehringer Ingelheim, Daiichi Sankyo, Bayer, Bristol Myers Squibb, and Pfizer Japan. T. Yamaguchi acted as an Advisory Board member of Daiichi Sankyo and received remuneration from Daiichi Sankyo and Bristol Myers Squibb. S. Teramukai received research funding from Nippon Boehringer Ingelheim and remuneration from Daiichi Sankyo, Sanofi, Takeda, Chugai Pharmaceutical, Solasia Pharma, Bayer, Sysmex, Nipro, NapaJen Pharma, Gunze, Kaneka, Kringle Pharma and Atworking. T. Kimura, Y. Morishima, and A. Takita are employees of Daiichi Sankyo. H. Inoue received remuneration from Daiichi Sankyo, Bristol Myers Squibb, and Nippon Boehringer Ingelheim.

## Supplementary Material


