# Supplementary material for: Home Blood Pressure Can Predict the Risk for Stroke/Bleeding Events in Elderly Patients With Nonvalvular Atrial Fibrillation From the ANAFIE Registry
Source: Hypertension. 2022 Oct 19;79(12):2696–705. doi: 10.1161/HYPERTENSIONAHA.122.19810 (PMC9640260; doi:10.1161/HYPERTENSIONAHA.122.19810)
Supplement: Supplementary file 1 [file hyp-79-2696-s001.pdf]

Home Blood Pressure Can Predict the Risk for Stroke/Bleeding Events in Elderly Patients with Non-Valvular Atrial Fibrillation from the ANAFIE Registry

Short title: Home BP and stroke/bleeding in elderly AF patients

Kazuomi Kario, MD, PhD[1]; Naoyuki Hasebe, MD, PhD[2], Ken Okumura, MD, PhD[3]; Takeshi Yamashita, MD, PhD[4]; Masaharu Akao, MD, PhD[5]; Hirotsugu Atarashi, MD, PhD[6]; Takanori Ikeda, MD, PhD[7]; Yukihiro Koretsune, MD, PhD[8]; Wataru Shimizu, MD, PhD[9]; Shinya Suzuki, MD, PhD[4]; Hiroyuki Tsutsui, MD, PhD[10]; Kazunori Toyoda, MD, PhD[11]; Atsushi Hirayama, MD, PhD[12]; Masahiro Yasaka, MD, PhD[13]; Takenori Yamaguchi, MD, PhD[11]; Satoshi Teramukai, PhD[14]; Tetsuya Kimura[15]; Yoshiyuki Morishima, PhD[15]; Atsushi Takita[15]; and Hiroshi Inoue, MD, PhD[16]

[1] Jichi Medical University, Tochigi, Japan

[2] Asahikawa Medical University, Hokkaido, Japan

[3] Saiseikai Kumamoto Hospital Cardiovascular Center, Kumamoto, Japan

[4] The Cardiovascular Institute, Tokyo, Japan

[5] National Hospital Organization Kyoto Medical Center, Kyoto, Japan

[6] AOI Hachioji Hospital, Tokyo, Japan

[7] Toho University Faculty of Medicine, Tokyo, Japan

[8] National Hospital Organization Osaka National Hospital, Osaka, Japan

[9] Nippon Medical School, Tokyo, Japan

[10] Kyushu University, Fukuoka, Japan

[11] National Cerebral and Cardiovascular Center, Osaka, Japan

[12] Osaka Police Hospital, Osaka, Japan

[13] National Hospital Organization Kyushu Medical Center, Fukuoka, Japan

[14] Kyoto Prefectural University of Medicine, Kyoto, Japan

[15] Daiichi Sankyo, Tokyo, Japan

[16] Saiseikai Toyama Hospital, Toyama, Japan

Corresponding author

Kazuomi Kario, MD, PhD, FACP, FACC, FAHA, FESC

Division of Cardiovascular Medicine, Jichi Medical University School of Medicine, 3311-1 Yakushiji, Shimotsuke, Tochigi 329-0498, Japan.

Tel: +81-0285-58-7538

E-mail: kkario@jichi.ac.jp

Trial registration: UMIN Clinical Trials Registry; UMIN000024006

SUPPLEMENTAL MATERIAL
for
Home Blood Pressure Can Predict the Risk for Stroke/Bleeding Events in Elderly Patients with Non-Valvular Atrial Fibrillation from the ANAFIE Registry

TABLE OF CONTENTS

**ANAFIE REGISTRY GROUP**	**Page 1**
**SUPPLEMENTARY METHODS**	**Page 9**
**SUPPLEMENTAL TABLES**	**Page 11**
**Table S1**	**Page 11**
**Table S2**	**Page 12**
**Table S3**	**Page 13**
**Table S4**	**Page 15**
**Table S5**	**Page 17**
**SUPPLEMENTAL FIGURES AND LEGENDS**	**Page 18**
**Figure S1**	**Page 18**
**Figure S2**	**Page 19**
**Figure S3**	**Page 20**
**Figure S4**	**Page 21**
**Figure S5**	**Page 22**

ANAFIE REGISTRY GROUP

Study organization

Steering committee

Inoue, Hiroshi	Saiseikai Toyama Hospital
Akao, Masaharu	National Hospital Organization Kyoto Medical Center
Atarashi, Hirotsugu	Nippon Medical School/ Minamihachioji Hospital
Hirayama, Atsushi	Osaka Police Hospital
Ikeda, Takanori	Toho University Omori Medical Center
Koretsune, Yukihiro	National Hospital Organization, Osaka National Hospital
Okumura, Ken	Saiseikai Kumamoto Hospital
Shimizu, Wataru	Nippon Medical School Hospital
Toyoda, Kazunori	National Cerebral and Cardiovascular Center
Tsutsui, Hiroyuki	Kyushu University Hospital
Yamaguchi, Takenori	National Cerebral and Cardiovascular Center
Yamashita, Takeshi	The Cardiovascular Institute
Yasaka, Masahiro	National Hospital Organization Kyushu Medical Center

Sub-study lead investigators

Akishita, Masahiro	University of Tokyo Hospital
Hasebe, Naoyuki	Asahikawa Medical University Hospital
Kario, Kazuomi	Jichi Medical University Hospital
Mizokami, Yuji	University of Tsukuba Hospital
Nagata, Ken	Yokohama General Hospital
Nakamura, Masato	Toho University Ohashi Medical Center
Terauchi, Yasuo	Yokohama City University Hospital
Yamamoto, Takatsugu	Teikyo University Hospital

Event adjudication committee

Fujimoto, Shigeru	Jichi Medical University Hospital
Hagiwara, Nobuhisa	Tokyo Women's Medical University Hospital
Hasegawa, Yasuhiro	St. Marianna University School of Medicine Hospital
Kawai, Yoko	Sanno Hospital
Kawana, Masatoshi	Tokyo Women's Medical University Hospital
Mizuno, Kyoichi	Mitsukoshi Health and Welfare Foundation

Statistical analysis officer

Teramukai, Satoshi	University Hospital, Kyoto Prefectural University of Medicine

Regional lead investigators

Abe, Koji	Okayama University
Abe, Yoshihisa,	Research Institute for Brain and Blood Vessels-Akita
Akasaka, Takashi	Wakayama Medical University Hospital
Ako, Junya	Kitasato University Hospital
Aonuma, Kazutaka	Cardiology Department, Institute of Clinical Medicine, Faculty of Medicine, University of Tsukuba
Asakawa, Tetsuya	Yamanashi Kosei Hospital
Daida, Hiroyuki	Juntendo University Hospital

Doi, Atsushi	Osaka University Hospital
Fukatani, Masahiko	Chikamorikai Chikamori Hospital
Fukumoto, Yoshihiro	Kurume University Hospital
Fukunami, Masatake	Osaka General Medical Center (Kyuseiki Sogo Iryo C)
Furukawa, Yutaka	Kobe City Medical Center General Hospital
Harada, Kazumasa	Tokyo Metropolitan Geriatric Hospital
Hasebe, Naoyuki	Asahikawa Medical University Hospital
Hashimoto, Yoichiro	Kumamoto City Hospital
Hata, Takashi	Shizuoka City Shimizu Hospital
Hiasa, Yoshikazu	Tokushima Red Cross Hospital
Higaki, Jitsuo	Minami-Matsuyama Hospital
Hirano, Teruyuki	Kyorin University Hospital
Hirao, Kenzo	Tokyo Medical And Dental University, Medical Hospital
Hirata, Kouichi	Dokkyo Medical University Hospital
Hiroshima, Kenichi	Kokura Memorial Hospital
Hokin, Kiyohiro	Hokkaido University Hospital
Horinaka, Shigeo	Dokkyo Medical University Hospital
Iguchi, Yasuyuki	Jikei University Hospital
Inden, Yasuya	Nagoya University Hospital
Ishikawa, Toshiyuki	Yokohama City University Hospital
Ito, Hiroshi	Okayama University
Ito, Masaaki	Mie University Hospital
Itsuro, Morishima	Ogaki Municipal Hospital
Kaneko, Yoshiaki	Gunma University Hospital
Katahira, Yoshiaki	Katta General Hospital
Kihara, Yasuki	Hiroshima University Hospital
Kimura, Kazumi	Nippon Medical School Hospital
Kitagawa, Kazuo	Tokyo Women's Medical University Hospital
Kitamura, Kazuo	University of Miyazaki Hospital
Kitaoka, Hiroaki	Kochi Medical School Hospital
Kitazono, Takanari	Kyushu University Hospital
Kobayashi, Shiro	Nippon Medical School Chiba Hokusoh Hospital
Kobayashi, Yoichi	Showa University School of Medicine
Kobayashi, Yoshinori	Tokai University Hachioji Hospital
Komuro, Issei	University of Tokyo Hospital
Kubota, Isao	Yamagata University School of Medicine
Kusano, Kengo	National Cerebral and Cardiovascular Center
Kuwahara, Koichiro	Shinshu University School of Medicine
Maekawa, Yuichiro	Hamamatsu University Hospital
Maemura, Koji	Nagasaki University Hospital
Masuyama, Tohru	Hyogo College of Medicine Hospital
Matoba, Satoaki	University Hospital Kyoto Prefectural University of Medicine
Matsumoto, Masayasu	Sakai City Medical Center
Matsuoka, Hideki	NHO Kagoshima Medical Center
Mihara, Ban	Mihara Memorial Hospital
Minamino, Tetsuo	Kagawa University Hospital
Minamino, Tohru	Niigata University Medical & Dental Hospital
Minoru, Horie	Shiga University of Medical Science
Mitamura, Hideo	KKR Tachikawa Hospital

Miura, Tetsuji	Sapporo Medical University Hospital
Miyauchi, Yasushi	Nippon Medical School Chiba Hokusoh Hospital
Momomura, Shinichi	Jichi Medical University Saitama Medical Center
Morino, Yoshihiro,	Iwate Medical University Hospital
Nakazato, Yuji	Juntendo University Urayasu Hospital
Ogawa, Masahiro	Fukuoka University Hospital
Ogawa, Satoshi	Ogawa Satoshi Clinic
Oisi, Mitsuru	Kagoshima University Hospital
Okada, Yasushi	NHO Kyushu Medical Center
Okishige, Kaoru	Yokohama City Minato Red Cross Hospital
Otsuki, Toshiho	Kindai University Hospital
Oya, Yusuke	University Hospital, University of the Ryukyus
Saito, Yoshihiko	Nara Medical University Hospital
Sakata, Yasushi	Osaka University Hospital
Sata, Masataka	Tokushima University Hospital
Shigenobu, Bando	Kagawa Prefectural Shirotori Hospital
Shizuta, Satoshi	Kyoto University Hospital
Suzuki, Michiyasu	Yamaguchi University Hospital
Tada, Hiroshi	University of Fukui Hospital
Takagi, Masahiko	Kansai Medical University Medical Center
Takahashi, Naohiko	Oita University Hospital
Takatsuki, Seiji	Keio University Hospital
Takizawa, Shunya	Tokai University Hospital
Tanabe, Kazuaki	Shimane University Hospital
Tanahashi, Norio	Saitama Medical University International Medical Center
Tasaka, Hiroshi	Kurashiki Central Hospital (Ohara HealthCare Foundation)
Terayama, Yasuo	Iwate Medical University Hospital
Tomimoto, Hidekazu	Mie University Hospital
Tomita, Hirofumi	Hirosaki University Hospital
Tsujino, Akira	Nagasaki University Hospital
Tsujita, Kenichi	Kumamoto University Hospital
Usuda, Kazuo	Toyama Prefectural Central Hospital
Watanabe, Eiichi	Fujita Health University Hospital
Yagita, Yoshiki	Kawasaki Medical School Hospital
Yakushiji, Yusuke	Saga University Hospital
Yamagishi, Masakazu	Kanazawa University Graduate School of Medicine
Yamaguchi, Shuhei	Shimane University Hospital
Yamamoto, Kazuhiro	Tottori University Hospital
Yamamoto, Yasumasa	Kyoto Katsura Hospital
Yamashina, Akira	Tokyo Medical University Hospital
Yano, Masafumi	Yamaguchi University Hospital
Yasuchika, Takeishi	Fukushima Medical University Hospital
Yasujima, Minoru	Hirosaki Stroke and Rehabilitation Center
Yoshio, Kobayashi	Chiba University Hospital

Study Secretariat

Yamashita, Takeshi	The Cardiovascular Institute
Suzuki, Shinya	The Cardiovascular Institute
Matsuda, Kazumi	The Cardiovascular Institute, CVI-ARO

Ishikawa, Hiroyuki	The Cardiovascular Institute, CVI-ARO

Participating centers in this sub-cohort study

Hokkaido

Asahikawa Medical University Hospital, Asahikawa Kosei General Hospital, Tonan Hospital, Rumoi City Hospital, Tada Naika, Akutsu Naika Iin, Hirasawa Cardiovascular Medicine Clinic, Kawakita Cardiovascular Medicine Clinic, Aishinkan Aishin Circulatory Organ Clinic, Nayoroshi Furen National Health Insurance Clinic, Miyazawa Cardiovascular Medicine Clinic, Fukagawa Municipal Hospital, Ebetsu City Hospital, Nayoro City General Hospital, Shinwakai Asahikawa Rehabilitation Hospital, Engaru-Kosei General Hospital, Keiyukai Yoshida Hospital, Miyanomori Memorial Hospital, Horada Naika Clinic, Ono Clinic, Tsukamoto Heart & Circulation Clinic, Kikusui Naika Junkanki Clinic, Nakamura Memorial Hospital

Aomori

Sato Naika Shonika Toriage Iin, Hachinohe Red Cross Hospital

Iwate

Odori Kamata Medicine Clinic, Emura Clinic, Shiroto Clinic, Oriso Internal Medicine Cardiology Clinic, Saien Cardiovascular Medicine Clinic, Kyoseikai Matsuzono Second Hospital, Iwate Medical University Hospital, Iwate Prefectural Kuji Hospital, Kitakami Saiseikai Hospital, Iwate Prefectural Ninohe Hospital

Miyagi

Matsuo Kenko Clinic, Hirasawa Medical Clinic, Japanese Red Cross Ishinomaki Hospital

Akita

Suzukiichiro Clinic

Yamagata

Yamanobe Fujita Clinic, Nihei Clinic, Aoyama Medical Clinic, Kitamurayama Hospital, Yamagata University Hospital, Nitobe Clinic, Nihonkai General Hospital, Medical Office Tachibana, Yamaguchi Heart Clinic, Hayasaka Internal Medicine Cardiovascular Clinic, Yamagata Prefectural Central Hospital, Tachibana Internal Medicine & Cardiology Clinic, Okitama Public General Hospital, Tsunoda Internal Medicine Clinic, Shinoda General Hospital, Miyuki-kai Hospital, Tohoku Central Hospital for Public School Teachers

Fukushima

Tani Clinic, Hirosaka Cardiology Medical Clinic

Ibaraki

Gyokushinkai Kashima Heart Clinic, Keijukai Itabashi Clinic, Kinu Medical Association Hospital, Tokushukai Koga General Hospital

Tochigi

NHO Tochigi Medical Center, Kawaguchi Clinic, Tokushinkai Moka Hospital, Dokkyo Medical University Nikko Medical Center, Jichi Medical University Hospital

Gunma

Seta Clinic, Yoshida Internal Medicine Clinic, Ryokushinkai Hosoya Iin, Takei Internal Medicine Cardiology, Tsurugaya Hospital, Flash Hospital, Mihara Memorial Hospital, Gunma University

Hospital, Fujioka General Hospital, Cardiovasular Hospital of Central Japan, NHO Takasaki General Medical Center, Public Tomioka General Hospital

Saitama

Kuwanomikai Kuwanomihongo Clinic, Keyaki Iin, Mizuno Internal Medical Clinic, Genkikai Ayumi Clinic, Kenjinkai Mashiko Hospital, Seijunkai Imanari Iin, Hiki Iin, Nakada Naika, Arai Clinic, Minamikoshigaya Kenshinkai Clinic, Dokkyo Medical University Saitama Medical Center, Shibuya Clinic, Sanyukai Saino Clinic, Shimizu Clinic Fusa, Shokokai Sanai Hospital

Chiba

Nippon Medical School Chiba Hokusoh Hospital, Chiba University Hospital, Yu Clinic, Yurinoki Clinic, Yanagisawakai Yanagisawa Iin, Juzenkai Tateyama Junkankinaika Geka, Hotchi Clinic, Kenkikai Kikuma Clinic, Seirei Sakura Citizen Hospital

Tokyo

Nihon University Itabashi Hospital, Jikei University Hospital, South Tokyo Heart Clinic, Uchiyama Clinic, Miyazaki Clinic, Murakami Iin, Kenkokan Suzuki Clinic, Kamada Clinic, Kenseikai Kobayashi Naika Clinic, Koyukai Oedo Orthopedic Surgery And Internal Medicine Clinic, Nikaidokai Kurosawa Clinic, Heiseikai Kambara Clinic, Otaki Cardiology Clinic, Fukagawa Gatharia Clinic, Tama Nagayama Jin Naika Clinic, Kenshinkai Minamino Cardiovascular Hospital, Kojikai Nakadai Iin, Shibuya Dogenzaka Clinic, Kitazumi Clinic, Gakuen Heart Clinic, Furukawa Naika Clinic, Shibamoto Clinic, Shozawa Clinic, JCHO Tokyo Yamate Medical Center, Nishisugamo Sato Wataru Naika Clinic, Higashi Tateishi Medical Clinic, Tsuboi Clinic, Nitobe Memorial Nakano General Hospital, Kishi Hospital, Kamiyama Iin, Sassa General Hospital, Senjuchuo Shinryojo, Yumino Heart Clinic, Okuda Clinic, Moritanikai Moritani Medical Clinic, Nishizawa Medical Clinic, Junkoukai Koto Hospital, Tenjinmae Clinic, Nerima General Hospital, Hosoda Clinic, Shinagawa Clinic

Kanagawa

Kunishima Clinic, Kanemoto Naika Junkankika Clinic, Sakura T's Clinic, Fujikura Clinic, Mineki Naika Clinic, Hayashi Clinic, Orange Clinic, Kanagawa Cardiovascular Medicine, Numata Naika Kokyukika, Sawano Clinic, Niizuma Clinic, Nishiyama Icho Junkanki Geka Iin, Kawata Clinic, Tsuchiya Clinic, Kaiseikai Kita Shin Yokohama Internal Medicine Clinic, Kamakura Yukinoshita Clinic, Idogaya-Naika Clinic, Nakanoshima Kitaguchi Kogawa Clinic, Kamegaya Clinic, Kimura Clinic, Kikuchi Clinic, Sohken Yamamoto Clinic, Yamato Municipal Hospital, St. Marianna University School of Medicine Hospital, Kitasato University Hospital, Odawara Cardiovascular Hospital, Ozawa Hospital, Kojima Clinic

Niigata

Kaetsu Clinic, Maeda Naika Iin

Toyama

Miwa Naika Clinic

Ishikawa

Handa Naika Clinic, Owari Cardiovascular & Diabetes Clinic, Japanese Red Cross Kanazawa Hospital, Junseikai Hakomiya Clinic

Fukui

Keiyukai Taga Naika Junkankika Iin, Meihokai Meiho Clinic, Tachibana Clinic, Arai Clinic, University of Fukui Hospital, Hayashi Hospital, Fukui Prefectural Hospital

Yamanashi

Kanoiwa General Hospital, Kuroda Clinic

Nagano

Hoden Clinic, Kimura Internal Medicine Clinic, Shinshu University School of Medicine

Gifu

Wakeikai Sugishita Iin, Igenkai Hui Ishiguro Clinic, Niimi Clinic, Asano Naika, Sunomata Clinic, Seijinkai Kawade Clinic, Takayama Red Cross Hospital, Ogaki Municipal Hospital

Shizuoka

JA-Shizuoka Enshu Hospital, Hamamatsu University Hospital, Kikugawa General Hospital

Aichi

Sugiyama Internal Medicine Clinic, Takezawa Clinic, Yoshida Clinic Internal & Cardiology, Okuda Naika Clinic, Iseki Clinic, Clinic Kakehashi, Suezawa Iin, Yasui Clinic, Daiyukai General Hospital, Tosei General Hospital, Nagoya Ekisaikai Hospital, Osugi-iin Medical Clinic

Mie

Tone Clinic, Nakamura Heart Clinic, Mie Prefectural General Medical Center, Mie University Hospital, Suzuka General Hospital

Shiga

Inagaki Heart Clinic

Kyoto

Inoue Cardiology Clinic, Takenaka Clinic, Kyoto Shimizu Medicare System Kyoto Rehabilitation Hospital, Rakuwakai Otowa Hospital, Higo Naika Iin

Osaka

JOHAS Osaka Rosai Hospital, NTT West Osaka Hospital, Wakaki Clinic, Sakai Medical Clinic, Fukuda Clinic, Yuwakai Sekine Iin, Tsurugaoka Azuma Clinic, Hirose Clinic, Murao Shinryojo, Matsushita Medical Clinic, Nakamura Medical Clinic, Nogi Internal Medicine Clinic, Koyokai Komori Medical Clinic, Fujita Naika・Nakano Naika, Osaka General Hospital of West Japan Railway Company, Sugimura Naika Clinic, Hamaguchi Iin, Japanese Red Cross Osaka Hospital

Hyogo

Hyogo College of Medicine Hospital, Nakata Naika Clinic, Hanakawa Clinic, Ueda Heart Clinic, Takeuchi Clinic, JOHAS Kobe Rosai Hospital, Kondo Naika Iin, Tsumura Cardiovascular Medicine Clinic, Hokudan Clinic, Miki Naika, Oya Iin, Shindo Clinic, Shinko Hospital, Katsuya Clinic, Okinawa Tokushukai Kobe Tokushukai Hospital

Nara

Enomoto Clinic

Wakayama

Junseikai Kitajimabashi Clinic, Yaeikai Yayoi Medical Clinic, Michiura Clinic, Komizo Clinic

Tottori

Sakaemachi Clinic

Shimane

Honda Clinic, Kawakami Clinic, Kimachi Clinic, Heisei Memorial Hospital

Okayama

Okayama Eastern Neurosurgery Tobi Clinic, Kenhoukai Sasaki Clinic, Yuhara Internal Clinic, Yasuda Internal Medicine Clinic, Kawahara Internal Medicine Clinic, Watanabe Hospital, Osakada Iin, NHO Okayama Medical Center

Hiroshima

Kunpukai Tanabe Clinic, Juntenkai Teshima Clinic, Clear Yakeyama Clinic, Seijinkai Murakami Internal Medicine Cardiology Iin, NHO Higashihiroshima Medical Center, Fukuyama Cardiovascular Hospital, Ninomiya Clinic

Yamaguchi

Yamaguchi University Hospital, Tojukai Ito Naika Iin, Yamaguchi Prefecture Grand Medical Center, Ryoke Clinic, Tokushinkai Ebihara Cardiovascular Internal Medicine, Anno Cardiovascular Clinic, Ogori Daiichi General Hospital

Tokushima

NHI Katsuura Hospital, Ota Shinryojo, Tokushima Red Cross Hospital, Minami Hospital, Rimuzu Tokushima Clinic

Kagawa

Hasegawa Outpatients Clinic for Cardiovascular Disease, Kunishige Makoto Clinic, Mitsu Heart Clinic, Olive Takamatsu Medical Clinic

Ehime

Tsubaki Clinic, Soyokaze Cardiovascular Medicine and Diabetes Care, Katagi Neurological Surgery, Takase Naika Clinic, Izumikawa Clinic, Ishite Matsumoto Internal Medicine Cardiology Clinic, Ehime Prefectural Central Hospital, Matsuyama Shimin Hospital

Kochi

Nakayama Clinic, Aoki Clinic, Clinic Hiroto, Nishino Clinic, Susaki Kuroshio Hospital

Fukuoka

Onga Hospital, Fukuda Cardiovascular Clinic, Katsuki Naika Clinic, Karinkai Murakamikarindoh Hospital, Sakurazaka Circulatory Clinic, Nakata Clinic, Yoshinaga Neurosurgical Clinic, Ninomiya Iin, Homma Clinic, Sakaue Clinic, Yamamoto Internal Medicine & Gastroenterology, NHO Fukuoka Higashi Medical Center, Yanagawa Hospital, Sasaguri Hospital, Toseikai Goto Clinic, Kurume University Medical Center, Steel Memorial Yawata Hospital, Hoseikai Takayama Hospital, Fujiwara Neurosurgical Clinic, Tomita Naika Junkankika, Kyushu Central Hospital of the Mutual Aid Association of Public School Teachers, Shin Komonji Hospital

Saga

Moroe Clinic

Nagasaki

Meiwakai Izaki Clinic, Seiyukai Mori Tsutomu Naika, Oka Clinic

Kumamoto

NHO Kumamoto Medical Center, Maki Naika Junkankika Iin, Aso Medical Center, Ueki Imafuji Clinic, Seiwakai Suizenji Touya Hospital, Saiseikai Kumamoto Hospital, Miyagi Junkanki Naika, Kumamoto Kinoh Hospital

Oita

Akeno Junkankinaika Clinic, NHO Oita Medical Center

Miyazaki

Yokota Naika, Keiwakai Kawano Iin, Eto Clinic, Seiwakai Fujiki Clinic, Ebino Sentoro Clinic, Hojukai Kawaida Clinic, Sonoda Hospital, Miyazaki Prefectural Nichinan Hospital, Miyazaki Seikyo Hospital

Kagoshima

Hiroshi Yamaguchi Clinic, Seijinkai Ikeda Hospital, Yukeikai Suetsugu Naika Junkankika, Shimozono Clinic, Eikokai Kumeda Naika Junkankinaika Clinic, Kashiwagikai Fukumoto Clinic, Arima Shinichi Clinic, Uemura Hospital, Imamura General Hospital

SUPPLEMENTARY METHODS

Exclusion Criteria

The main exclusion criteria were a definite diagnosis of mitral stenosis; an artificial heart valve replacement; very recent history (within 1 month prior to enrollment) of cardiovascular events, including stroke, myocardial infarction, cardiac intervention, heart failure requiring hospitalization, or any bleeding leading to hospitalization; or life expectancy <1 year.

Data Collection

For the present analysis, the data analyzed comprised patient demographics and background characteristics (disease history, comorbidities, and complications), H-BP measurements, laboratory values, clinical examination data, type of anticoagulant used, concomitant medications, and adverse events.

Statistical Analysis

A spline regression analysis was performed to investigate the relative risks of the net cardiovascular outcome and major bleeding in association with H-SBP. Prognostic factors were incorporated into the model, and the mean H-SBP value for the entire sub-cohort was used as a reference. The natural cubic spline was used for the H-SBP value, the number of knots was set to 5, and the quantile levels were set to 0.05, 0.275, 0.5, 0.725, and 0.95.

Definition of Events

Event	Time to event	Time to censoring (if no event occurs)
Stroke/ systemic embolism*	Time from the date of obtainment of informed consent to the first occurrence of “atherosclerotic, cardiogenic, lacunar, or unclassified cerebral infarction; hemorrhagic stroke (cerebral hemorrhage excluding traumatic type); hemorrhagic stroke (subarachnoid hemorrhage excluding traumatic type); unclassified cerebral hemorrhage, other stroke; or systemic embolism”	Time from the date of obtainment of informed consent to the last known alive date or date of death
Major bleeding	Time from the date of obtainment of informed consent to the first occurrence of “major bleeding”<br>Major bleeding was classified according to the International Society on Thrombosis and Haemostasis definition (including fatal bleeding, and/or symptomatic bleeding in a critical area or organ, such as intracranial, intraspinal, intraocular, retroperitoneal, intra-articular or pericardial, or intramuscular with compartment syndrome, and/or bleeding causing a fall in hemoglobin level of 20 g/L or more, or leading to transfusion of two or more units of whole blood or red cells).[21]	Time from the date of obtainment of informed consent to the last known alive date or date of death
Intracranial hemorrhage	Time from the date of obtainment of informed consent to the first occurrence of “cerebral hemorrhage, subarachnoid hemorrhage (traumatic), subdural/epidural hemorrhage, or other intracranial hemorrhage”	Time from the date of obtainment of informed consent to the last known alive date or date of death
Cardiovascular death	Time from the date of obtainment of informed consent to “death from cardiovascular causes”	From the date of obtainment of informed consent to the last known alive date or date of death for “death other than that for which cardiovascular death cannot be denied”
All-cause death	Time from the date of obtainment of informed consent to “death from all causes”	From the date of obtainment of informed consent to the last known alive date

*Primary endpoint.

SUPPLEMENTAL TABLES

Table S1. Patient Characteristics in the Entire ANAFIE Registry Cohort and in the Home Blood Pressure Sub-Cohort

Characteristic	ANAFIE Registry N=32275	This sub-cohort N=4933
Age, years	81.5±4.8	81.4±4.8
Men	18482 (57.3)	2770 (56.2)
Body mass index, kg/m^2	23.3±3.6	23.4±3.6
Creatinine clearance, mL/min	48.6±22.0	49.1±17.1
<50 mL/min	14633 (45.3)	2180 (44.2)
Comorbidities	31400 (97.3)	4800 (97.3)
Hypertension	24312 (75.3)	3829 (77.6)
Dyslipidemia	13728 (42.5)	2132 (43.2)
Diabetes mellitus	8733 (27.1)	1278 (25.9)
Chronic kidney disease	6705 (20.8)	926 (18.8)
Cardiac diseases	19024 (58.9)	2725 (55.2)
Atrial fibrillation type		
Paroxysmal	13586 (42.1)	2052 (41.6)
Non-paroxysmal	18689 (57.9)	2881 (58.4)
CHA_2DS_2-VASc score	4.5±1.4	4.4±1.3
HAS-BLED score	1.9±0.9	1.8±0.8
Office systolic blood pressure, mmHg	127.4±17.0	128.4±17.2
Office diastolic blood pressure, mmHg	70.6±11.6	71.3±11.5
Antiplatelet agents	5704 (17.7)	827 (17.4)
Antihypertensives	22892 (70.9)	3658 (77.1)
Anticoagulants	29830 (92.4)	4590 (93.0)
Warfarin	8233 (25.5)	1092 (22.1)
Direct oral anticoagulant	21585 (66.9)	3494 (70.8)
Dabigatran	2347 (7.3)	357 (7.2)
Rivaroxaban	6403 (19.8)	1184 (24.0)
Apixaban	8045 (24.9)	1184 (24.0)
Edoxaban	4790 (14.8)	769 (15.6)

Data are n (%) or mean±standard deviation.

Table S2. Analyses of Akaike's Information Criterion and Schwarz Bayesian Information Criterion Calculated for the Following Two Models of Office (O) and Home (H) Blood Pressure (BP): "Model B: Confounders + O-SBP"; "Model C: Confounders + H-SBP"

Model	Net CV Outcome		Stroke/SEE		Major bleeding		Intracranial hemorrhage		All-cause deaths	
	AIC	BIC	AIC	BIC	AIC	BIC	AIC	BIC	AIC	BIC
A: confounders* only	2884.46	2994.62	1905.13	2001.20	1301.91	1383.49	986.10	1057.60	4843.73	4973.25
B: confounders + O-SBP (categorical)‡	2886.23	2999.54	1905.96	2004.78	1302.78	1386.68	988.00	1061.55	4845.23	4978.44
C: confounders + H-SBP (categorical)#	2879.57	2992.88	1903.67	2002.49	1290.96	1374.86	975.27	1048.82	4843.71	4976.93

*Included in the model as confounding variables were the same as in Table 2.

‡Office systolic blood pressure values were added as categorical variables (<160 mmHg/≥160 mmHg).

#Home systolic blood pressure values were added as categorical variables (<145 mmHg/≥145 mmHg).

AIC indicates Akaike's information criterion; BIC, Schwarz Bayesian information criterion; BP, blood pressure; CV, cardiovascular; H-SBP, home systolic blood pressure; OAC, oral anticoagulant; O-SBP, office systolic blood pressure; SEE, systemic embolic events

Table S3. Incidence per 100 Person-Years by Type of Hypertension: 1) Controlled; H-SBP <145 mmHg/O-SBP <160 mmHg, 2) Masked Hypertension; H-SBP ≥145 mmHg/O-SBP <160 mmHg, 3) White Coat Hypertension; H-SBP <145 mmHg/O-SBP ≥160 mmHg, 4) Sustained; H-SBP ≥145 mmHg/O-SBP ≥160 mmHg Hypertension

Event	Group	Number of patients	Number of events (%)		Incidence per 100 person-years (95% CI)	*P*-value*
Net CV outcome	Overall	4,933	172	(3.49)	1.88 (1.60–2.16)	
	Controlled	4,335	143	(3.30)	1.78 (1.48–2.07)	Ref
	Masked hypertension	395	23	(5.82)	3.19 (1.89–4.50)	0.009
	White coat hypertension	158	3	(1.90)	1.00 (0.00–2.12)	0.323
	Sustained hypertension	45	3	(6.67)	3.59 (0.00–7.65)	0.227
Stroke/SEE	Overall	4,933	115	(2.33)	1.25 (1.02–1.48)	
	Controlled	4,335	96	(2.21)	1.19 (0.95–1.42)	Ref
	Masked hypertension	395	16	(4.05)	2.20 (1.12–3.28)	0.022
	White coat hypertension	158	2	(1.27)	0.66 (0.00–1.58)	0.416
	Sustained hypertension	45	1	(2.22)	1.19 (0.00–3.53)	0.996
Major bleeding	Overall	4,933	76	(1.54)	0.82 (0.64–1.01)	
	Controlled	4,335	57	(1.31)	0.70 (0.52–0.88)	Ref
	Masked hypertension	395	14	(3.54)	1.93 (0.92–2.94)	<0.001
	White coat hypertension	158	2	(1.27)	0.66 (0.00–1.58)	0.938
	Sustained hypertension	45	3	(6.67)	3.59 (0.00–7.65)	0.006
Intracranial hemorrhage	Overall	4,933	57	(1.16)	0.62 (0.46–0.78)	
	Controlled	4,335	42	(0.97)	0.52 (0.36–0.67)	Ref
	Masked hypertension	395	13	(3.29)	1.79 (0.82–2.76)	<0.001

	White coat hypertension	158	1	(0.63)	0.33 (0.00–0.98)	0.661
	Sustained hypertension	45	1	(2.22)	1.20 (0.00–3.54)	0.407
All-cause death	Overall	4,933	299	(6.06)	3.22 (2.86–3.59)	
	Controlled	4,335	257	(5.93)	3.15 (2.77–3.54)	Ref
	Masked hypertension	395	33	(8.35)	4.48 (2.95–6.01)	0.057
	White coat hypertension	158	6	(3.80)	1.99 (0.40–3.58)	0.265
	Sustained hypertension	45	3	(6.67)	3.53 (0.00–7.53)	0.844

*The *P*-value of the incidence-rate ratio in the Poisson regression model.
CV indicates cardiovascular; H-SBP, home systolic blood pressure; O-SBP, office systolic blood pressure; SEE, systemic embolic events.

Table S4. Incidence per 100 Person-Years by Type of Hypertension: 1) Well Controlled; H-SBP <135 mmHg/O-SBP <140 mmHg, 2) Masked Hypertension; H-SBP ≥135 mmHg/O-SBP <140 mmHg, 3) White Coat Hypertension; H-SBP <135 mmHg/O-SBP ≥140 mmHg, 4) Sustained; H-SBP ≥135 mmHg/O-SBP ≥140 mmHg Hypertension

Event	Group	Number of patients	Number of events (%)		Incidence per 100 person-years (95% CI)	*P*-value*
Net CV outcome	Overall	4,933	172	(3.49)	1.88 (1.60–2.16)	
	Well controlled	2,943	94	(3.19)	1.72 (1.37–2.07)	Ref
	Masked hypertension	800	28	(3.50)	1.89 (1.19–2.59)	0.671
	White coat hypertension	672	23	(3.42)	1.82 (1.08–2.56)	0.814
	Sustained hypertension	518	27	(5.21)	2.82 (1.76–3.89)	0.024
Stroke/SEE	Overall	4,933	115	(2.33)	1.25 (1.02–1.48)	
	Well controlled	2,943	64	(2.17)	1.17 (0.88–1.46)	Ref
	Masked hypertension	800	17	(2.13)	1.14 (0.60–1.68)	0.921
	White coat hypertension	672	16	(2.38)	1.26 (0.64–1.88)	0.788
	Sustained hypertension	518	18	(3.47)	1.87 (1.01–2.74)	0.077
Major bleeding	Overall	4,933	76	(1.54)	0.82 (0.64–1.01)	
	Well controlled	2,943	37	(1.26)	0.67 (0.46–0.89)	Ref
	Masked hypertension	800	14	(1.75)	0.94 (0.45–1.43)	0.295
	White coat hypertension	672	9	(1.34)	0.70 (0.24–1.17)	0.904
	Sustained hypertension	518	16	(3.09)	1.66 (0.85–2.48	0.003
Intracranial hemorrhage	Overall	4,933	57	(1.16)	0.62 (0.46–0.78)	
	Well controlled	2,943	28	(0.95)	0.51 (0.32–0.70)	Ref
	Masked hypertension	800	10	(1.25)	0.67 (0.25–1.08)	0.464

	White coat hypertension	672	7	(1.04)	0.55 (0.14–0.95)	0.862
	Sustained hypertension	518	12	(2.32)	1.25 (0.54–1.95)	0.010
All-cause death	Overall	4,933	299	(6.06)	3.22 (2.86–3.59)	
	Well controlled	2,943	197	(6.69)	3.57 (3.07–4.07)	Ref
	Masked hypertension	800	46	(5.75)	3.05 (2.17–3.93)	0.332
	White coat hypertension	672	27	(4.02)	2.10 (1.31–2.90)	0.010
	Sustained hypertension	518	29	(5.60)	2.99 (1.90–4.07)	0.367

*The *P*-value of the incidence-rate ratio in the Poisson regression model.
CV indicates cardiovascular; H-SBP, home systolic blood pressure; O-SBP, office systolic blood pressure; SEE, systemic embolic events.

Table S5. Causes of Death

Cause of death	Total population	H-SBP <125 mmHg	H-SBP ≥125 to <135 mmHg	H-SBP ≥135 to <145 mmHg	H-SBP ≥145 mmHg
Total all-cause death	299	140	84	39	36
Fatal bleeding	18 (6.0)	7 (5.0)	3 (3.6)	2 (5.1)	6 (16.7)
Intracranial hemorrhage	7 (2.3)	3 (2.1)	1 (1.2)	0 (0)	3 (8.3)
Extracranial bleeding	11 (3.7)	4 (2.9)	2 (2.4)	2 (5.1)	3 (8.3)
Nonbleeding cardiovascular deaths	75 (25.1)	32 (22.9)	22 (26.2)	11 (28.2)	10 (27.8)
Ischemic stroke	16 (5.4)	7 (5.0)	5 (6.0)	2 (5.1)	2 (5.6)
Heart failure/cardiac arrest	27 (9.0)	13 (9.3)	5 (6.0)	6 (15.4)	3 (8.3)
Pulmonary embolism	1 (0.3)	0 (0)	1 (1.2)	0 (0)	0 (0)
Cardiac sudden death	18 (6.0)	6 (4.3)	8 (9.5)	3 (7.7)	1 (2.8)
Other cardiovascular death	13 (4.3)	6 (4.3)	3 (3.6)	0 (0)	4 (11.1)
Malignancy	50 (16.7)	27 (19.3)	17 (20.2)	2 (5.1)	4 (11.1)
Other	156 (52.2)	74 (52.9)	42 (50.0)	24 (61.5)	16 (44.4)
Infection	57 (19.1)	26 (18.6)	16 (19.0)	10 (25.6)	5 (13.9)
Hepatobiliary	1 (0.3)	0 (0)	1 (1.2)	0 (0)	0 (0)
Renal	9 (3.0)	5 (3.6)	2 (2.4)	1 (2.6)	1 (2.8)
Other	89 (29.8)	43 (30.7)	23 (27.4)	13 (33.3)	10 (27.8)

Data are n (%).

H-SBP indicates home systolic blood pressure.

SUPPLEMENTAL FIGURES AND LEGENDS

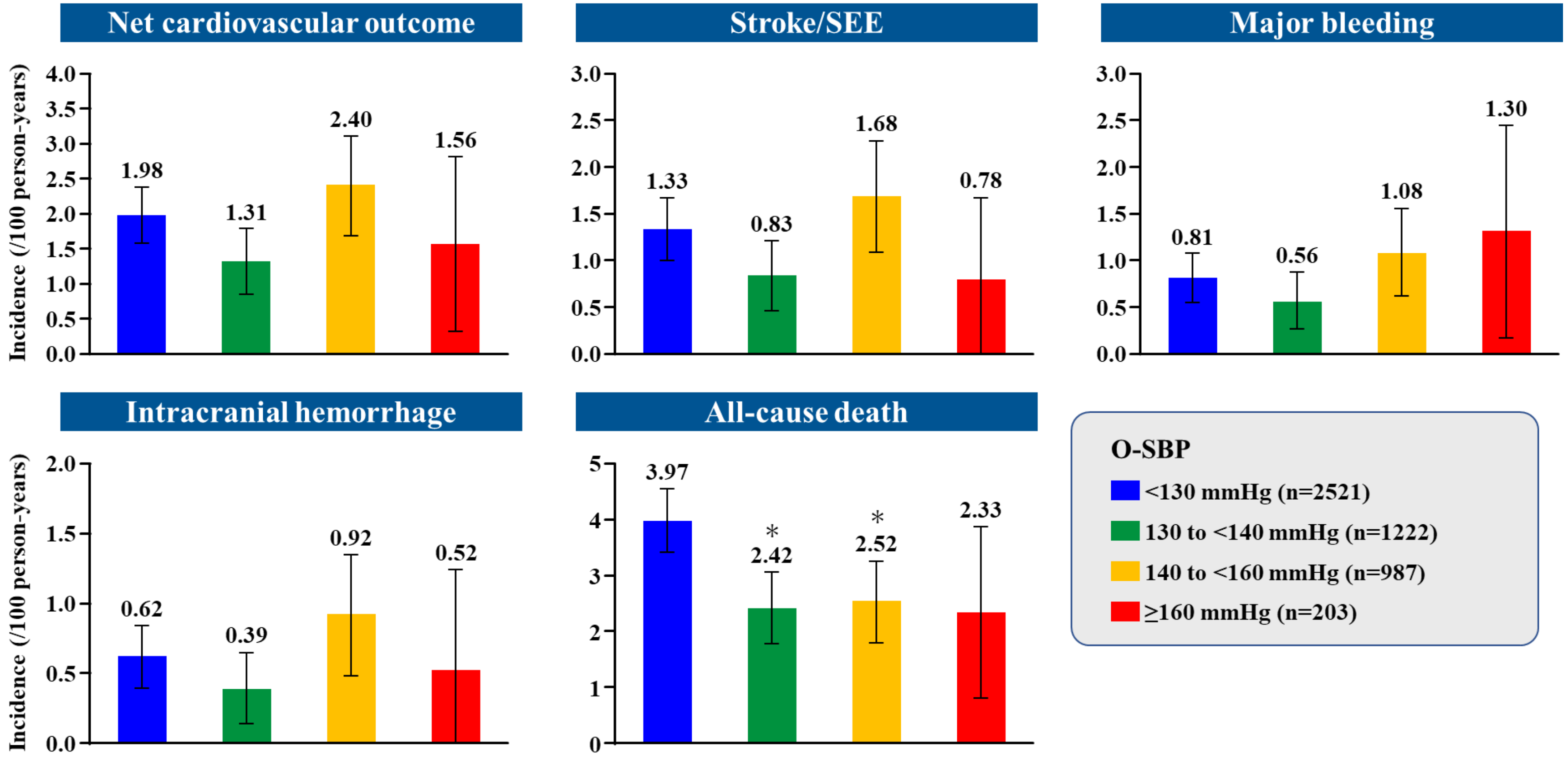

Figure S1. O-SBP and incidence rates of outcome events. **P*<0.05 versus O-SBP <130 mmHg
O-SBP indicates office systolic blood pressure; SEE, systemic embolic events.

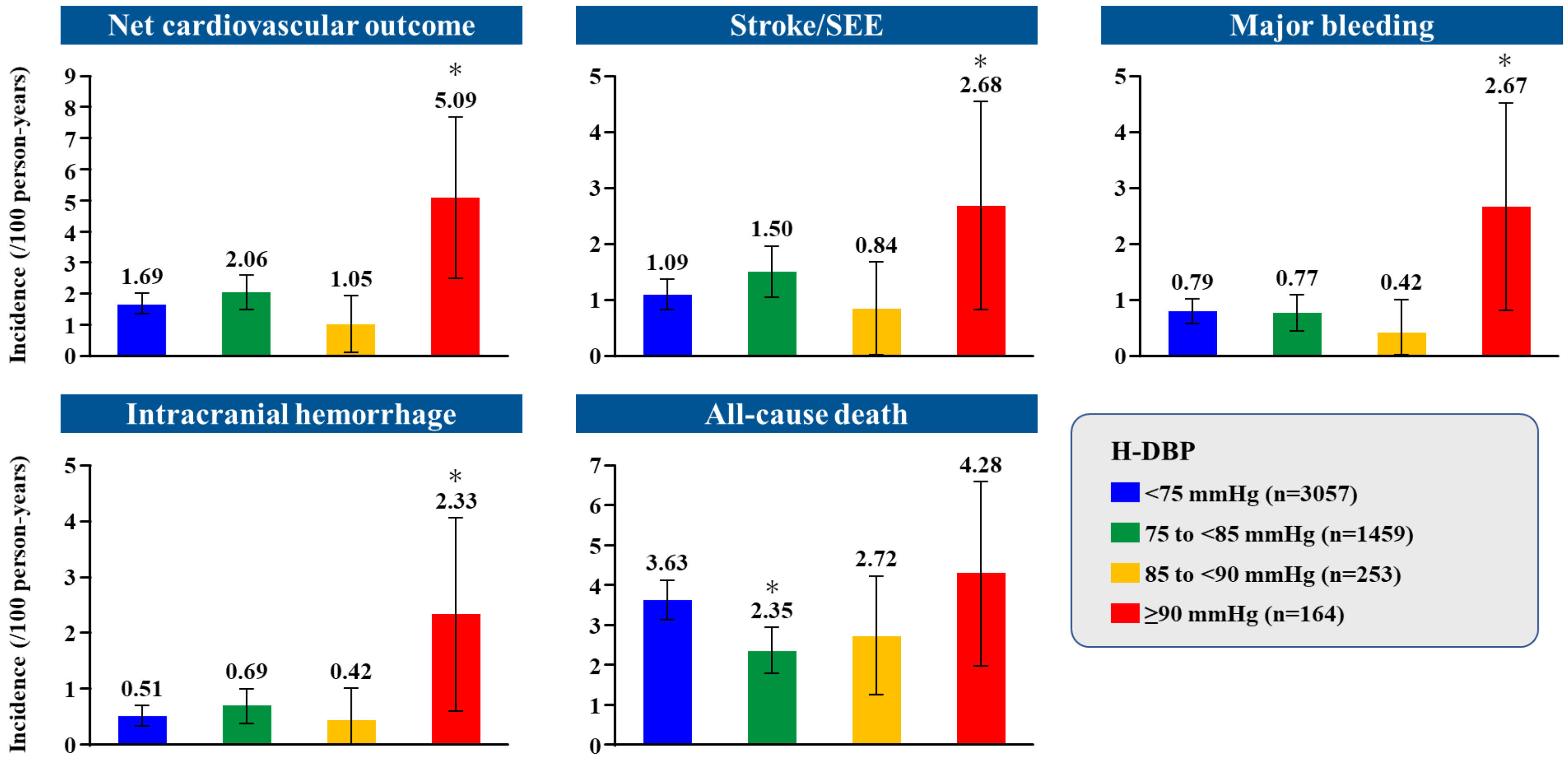

Figure S2. H-DBP and incidence rates of outcome events. *P<0.05 versus H-DBP <75 mmHg
H-DBP indicates home systolic blood pressure; SEE, systemic embolic events.

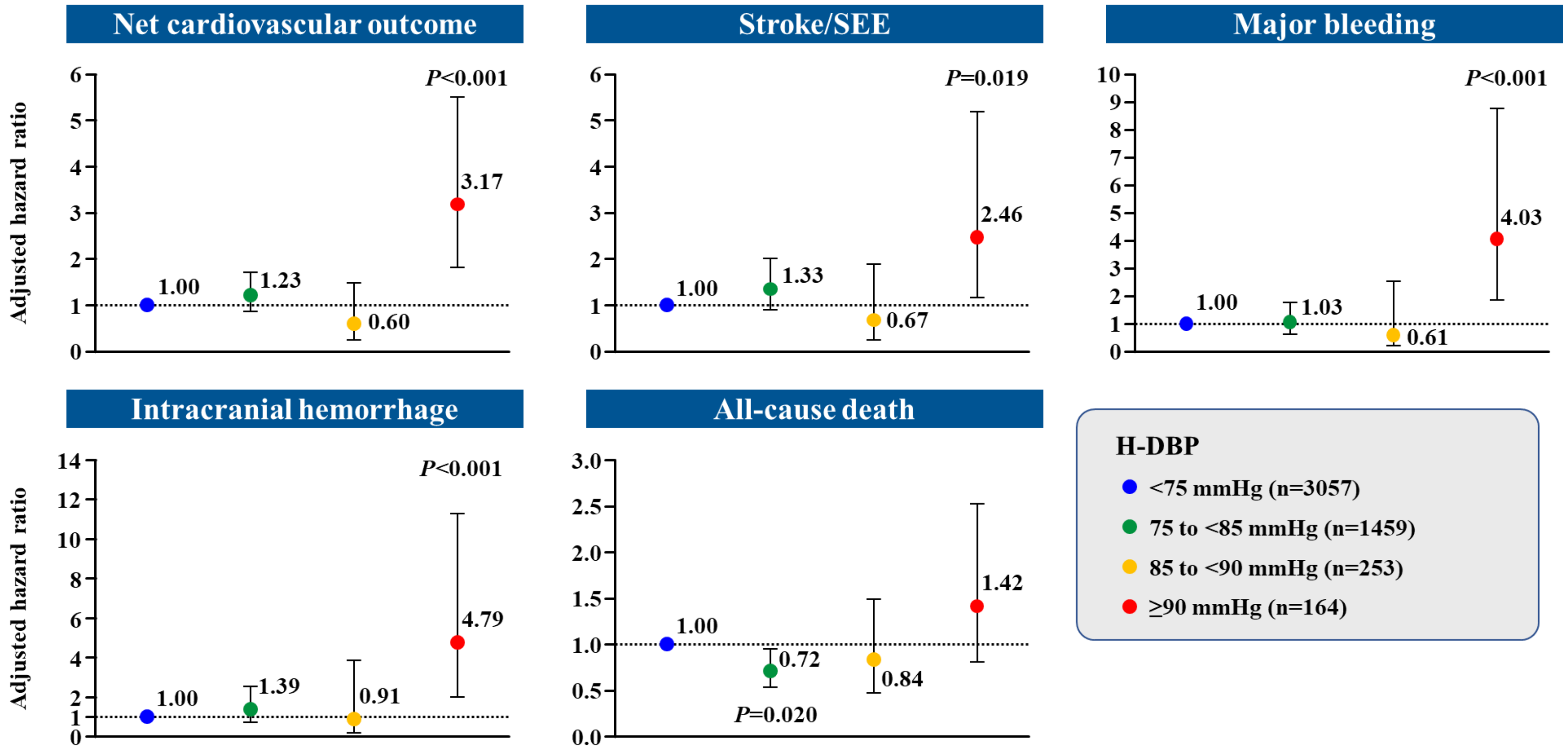

Figure S3. H-DBP and hazard ratio for outcome events.
H-DBP indicates home systolic blood pressure; SEE, systemic embolic events.

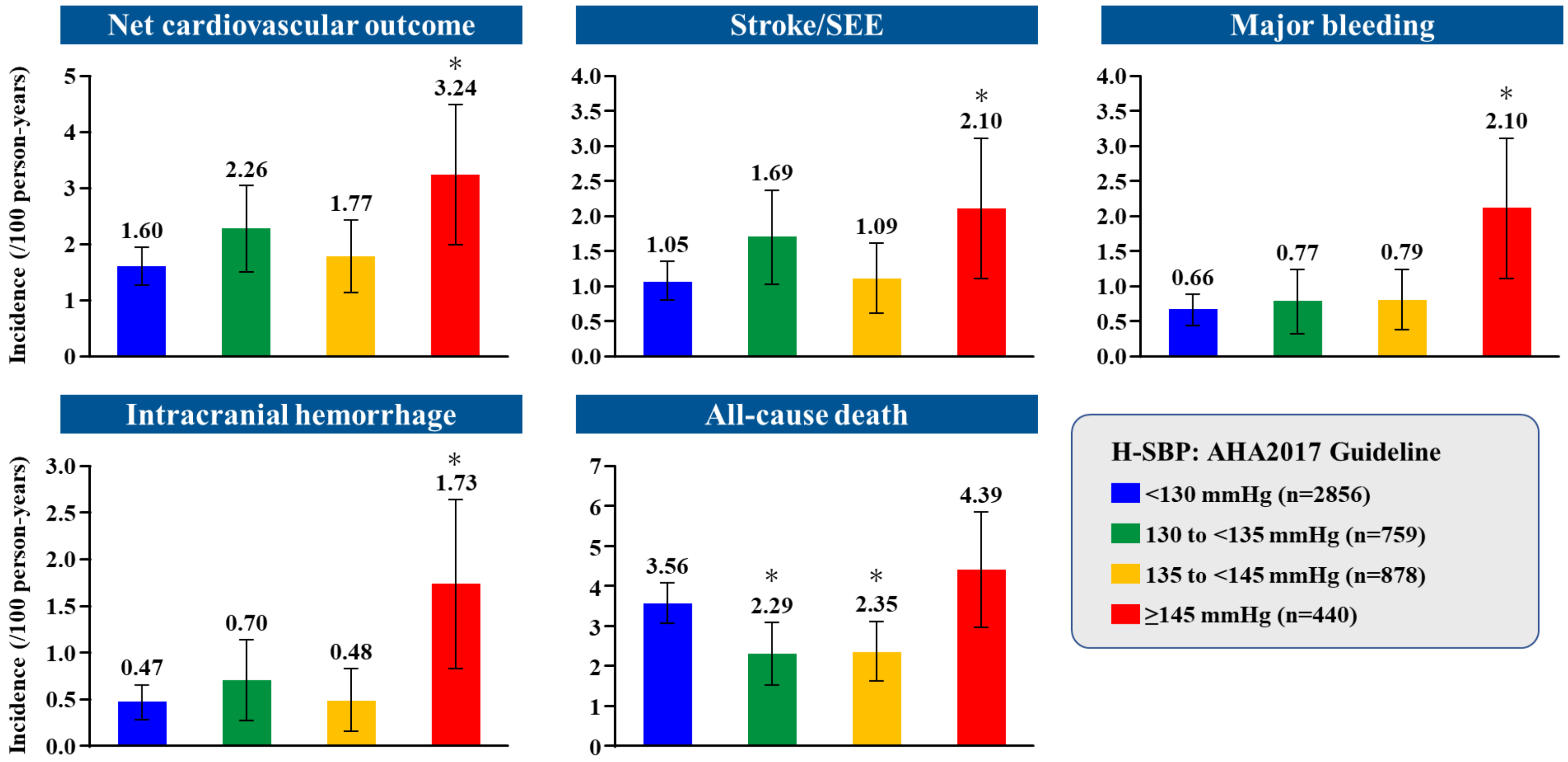

Figure S4. H-SBP (AHA 2017 Guideline) and incidence rates of outcome events.
H-SBP indicates home systolic blood pressure; SEE, systemic embolic events.

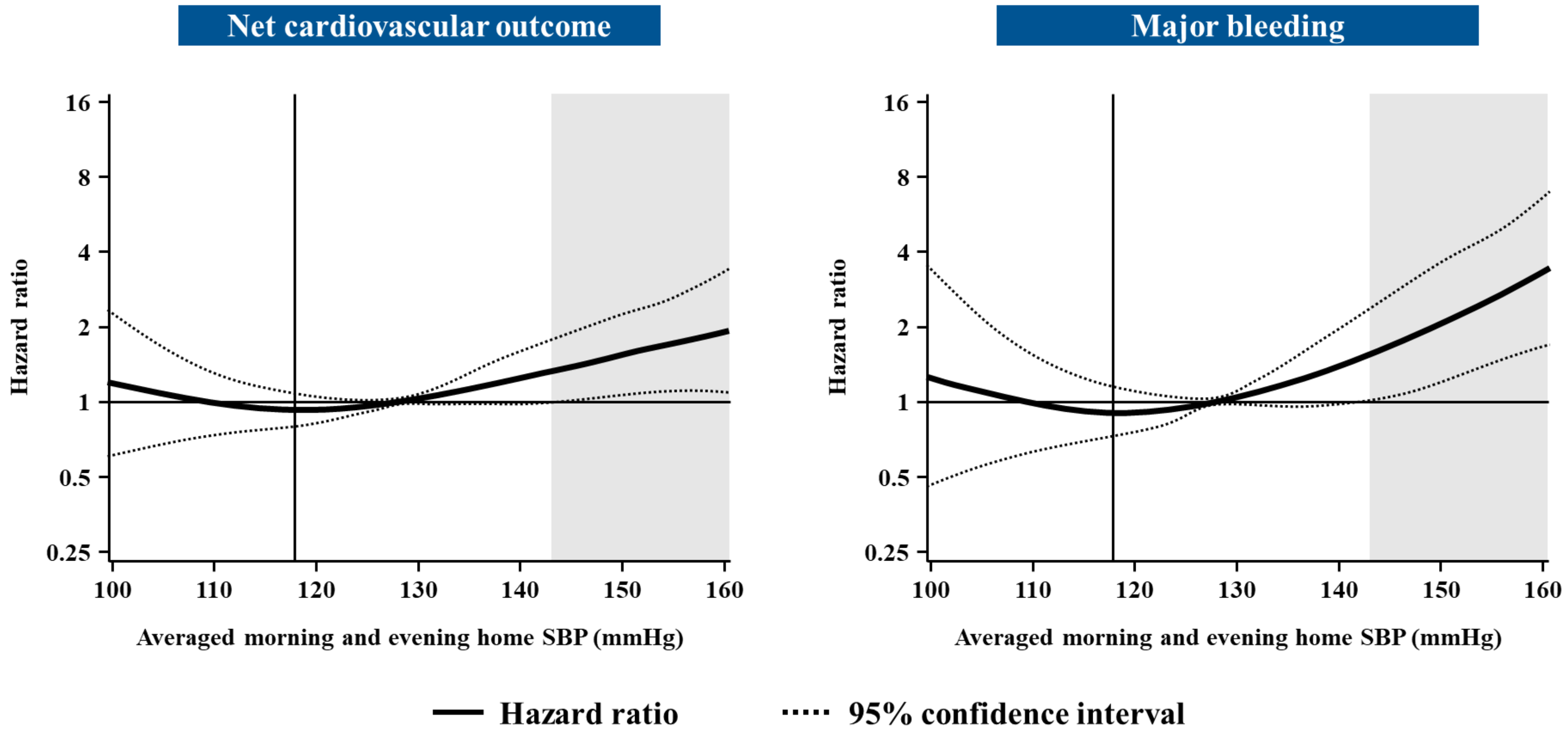

Figure S5. Spline regression analysis of relationships between H-SBP and hazard ratios for net cardiovascular outcome and major bleeding. The vertical line at 118 mmHg indicates minimum risk. The portion of each graph with gray shading represents the range of H-SBP values where the lower limit of the 95% CI is >1.
H-SBP indicates home systolic blood pressure.
